# Making the most of RNA-seq: Pre-processing sequencing data with Opossum for reliable SNP variant detection

**DOI:** 10.12688/wellcomeopenres.10501.2

**Published:** 2017-03-17

**Authors:** Laura Oikkonen, Stefano Lise

**Affiliations:** 1 Wellcome Trust Centre for Human Genetics, University of Oxford, Oxford, UK; 2 Centre for Evolution and Cancer, The Institute of Cancer Research, Sutton, UK

**Keywords:** RNA-seq, variant calling, SNP, software tools

## Abstract

RNA-seq (transcriptome sequencing) is primarily considered a method of gene expression analysis but it can also be used to detect DNA variants in expressed regions of the genome. However, current variant callers do not generally behave well with RNA-seq data due to reads encompassing intronic regions. We have developed a software programme called Opossum to address this problem. Opossum pre-processes RNA-seq reads prior to variant calling, and although it has been designed to work specifically with Platypus, it can be used equally well with other variant callers such as GATK HaplotypeCaller. In this work, we show that using Opossum in conjunction with either Platypus or GATK HaplotypeCaller maintains precision and improves the sensitivity for SNP detection compared to the GATK Best Practices pipeline. In addition, using it in combination with Platypus offers a substantial reduction in run times compared to the GATK pipeline so it is ideal when there are only limited time or computational resources available.

## Introduction

RNA-seq (transcriptome sequencing) ^1^ is routinely employed for gene expression analysis, but it can also be used to identify genomic variants in expressed regions alongside whole-exome (WES) and whole-genome sequencing (WGS). Recently, its potential in improving diagnostics was demonstrated in a clinical setting ^2^. However, since the prevalent variant calling pipelines have been designed specifically for DNA data, novel tools or modifications to the existing ones are needed for processing RNA-seq data. Detecting variants in lowly expressed genes, covered by only a few reads, poses strict demands on the precision and sensitivity of the method. Moreover, the method needs to be able to cope with intron-spanning RNA-seq reads.

A few pipelines for detecting SNPs in RNA-seq data have now been released to address these challenges. eSNV-detect by Tang *et al.* ^3^ employs a combination of mappers to overcome systematic errors of individual aligners, followed by variant calling with Samtools and Bcftools. SNPiR by Piskol *et al.* ^4^ relies on a single aligner (BWA) to map reads across splice junctions and filters heavily after variant calling done with GATK UnifiedGenotyper, at the cost of decreased sensitivity. Also the developers of GATK have released online their Best Practices for calling variants from RNA-seq data ( https://software.broadinstitute.org/gatk//guide/article?id=3891). All of them mix and match parts of older pipelines developed for DNA data processing in order to make sense of RNA-seq data. The benchmarking in these studies has not been done consistently, making it difficult to directly compare their performance.

Current state-of-the-art variant calling algorithms employ a haplotype-driven strategy to achieve higher accuracy. For example Platypus ^5^ performs a local *de novo* read assembly to generate candidate variants and reconstruct haplotypes. Variants are then called based on the estimated haplotypes. The approach works well on length scales of up to a few kilobases (typically up to 1.5–2 kb) but longer reads (e.g. reads mapping across large introns) would disrupt it. For this reason Platypus should not be run directly on RNA-seq data.

In this work, we have developed a software tool called Opossum ^6^ specifically to process and filter RNA-seq data and make it suitable for (haplotype-based) variant calling. No additional processing step (e.g. base quality recalibration) or filtering is required. The presence of splice junctions in RNA-seq data means that reads which have been mapped across splice junctions must be split to remove intronic parts which would otherwise disrupt variant calling. Now, after splitting, we would generally lose information of which new shorter reads originated from the same longer read. This, in turn, would mean that more base-changes would be ignored at the variant calling stage since typically bases are ignored from both ends of each read, and also the possible overlap of originally paired-end reads could not be detected any more. Opossum overcomes these issues by merging overlapping reads and by modifying the base qualities of bases at the ends of the original reads before splitting them. As a result, all information is already incorporated into the reads, and the variant caller can be run with minimal settings. Opossum can be used together with different aligners (TopHat ^7^, Star ^8^) and provides ways for adjusting for the peculiarities of each aligner. While it has been designed to work particularly with Platypus ^5^, Opossum can be used equally well with other variant callers such as GATK HaplotypeCaller ^9^. Our approach shows promising results, maintaining high precision and improving sensitivity in detecting SNP variant calls compared to the GATK Best Practices pipeline. As a reference, we have used the strongly validated GIAB (Genome in a Bottle) dataset ^10^.

## Methods

### Operation

Opossum ^6^ is a Python-based software, requiring Python 2.7 (or greater) along with Python packages Pysam v0.10.0 ( https://github.com/pysam-developers/pysam), itertools, argparse, os and sys. Pysam v0.10.0 wraps htslib-1.3, samtools-1.3 and bcftools-1.3 ^12^. Opossum has not been tested with the Python 3.X series.

As input, Opossum requires a position-sorted BAM file, which is then processed for variant calling. When running the program, the user should specify whether the input BAM file includes any soft clips ( *’SoftClipsExist’*, default=False). The user can also decide whether only properly paired reads should be considered ( *’ProperlyPaired’*, default=True) and what is the minimum acceptable mapping quality for a read pair ( *’MapCutoff’*, default=40). Note that in TopHat and Star, mapping qualities can only take a restricted set of values: from 0 to 3 if a read maps to multiple locations, 50 (TopHat) or 255 (Star) if a read is a uniquely mapped (In the SAM format specification, a value of 255 indicates that a mapping quality is not available. Opossum therefore reassigns to these reads a quality value of 50. Alternatively Star can be run with the option *’–outSAMmapqUnique 50’* to modify the value assigned to uniquely mapped reads). The precise *’MapCutoff’* value is therefore not important for these mappers as long as it is between 4 and 49. However, it could become relevant if Opossum is used in conjunction with other mappers e.g. HiSat2 ^13^ as quality scores can then take up a wider range of values.

Opossum output is a sorted and indexed BAM file on which SNP variant calling can be carried out with, e.g., Platypus with minimal settings since Opossum has already cleaned the data. By default, Platypus flags variants that do not fulfill all of its filtering criteria ^5^. These criteria have been designed to make the most out of DNA data. The same criteria can well be used with RNA-seq data if the user wants to maximize precision at the cost of sensitivity. However, if the user seeks a greater balance between precision and sensitivity, it would be advisable to include also variants flagged as ’badReads’, ’SC’, and ’Q20’ among the final variants.

### Implementation

Opossum starts by taking several quality control measures. It discards secondary alignments and reads that have a mapping quality lower than the cutoff specified by the user (via *’MapCutoff’*). Opossum also gets rid of reads in pairs that have been aligned in the same direction or are pointing outwards, and paired-end reads where the two reads have been mapped to different chromosomes.

Next, Opossum gets rid of read duplicates. Duplicates are defined as read pairs having identical 5’ coordinates and orientations. After duplicate reads have been collected, the primary read is chosen among the properly paired reads based on which pair has the highest sum of base qualities. Then the primary read is compared with each secondary read and modified to accommodate differences in the following way: If the primary and secondary reads have a base-wise discrepancy with a very low base quality (i.e. one or both reads have base quality of less than 10), then the higher-quality base is kept. If both base qualities are above 10, then the corresponding base quality in the primary read is set to zero to reflect the uncertainty involved. This differs from e.g. Picard MarkDuplicates ( https://broadinstitute.github.io/picard/command-line-overview.html#MarkDuplicates) which ignores read flags and does not modify primary reads. Single reads are discarded as duplicates if they have the same starting position as a paired-end read; otherwise, a primary read is chosen among the single read duplicates.

Opossum merges overlapping paired-end reads to avoid double-counting the overlapping part during variant calling. The user can specify whether overlapping paired-end reads having at least one base mismatch within the overlap region should be kept ( *’KeepMismatches’*, default=False). If they are kept and one of the reads has a very low-quality base at a mismatch position, then the higher-quality base is kept. Otherwise if both base qualities are above 10, then the corresponding base quality in the merged read is set to zero. Reads with intronic regions (denoted by *N* in the CIGAR string) are split to only keep the exonic parts, resulting in new, shorter reads. If the overlapping parts of reads in a pair have not been aligned to the same exons, the pair is discarded as the mapping cannot be trusted. The final, merged reads are always aligned on the forward strand.

**Figure 1.**
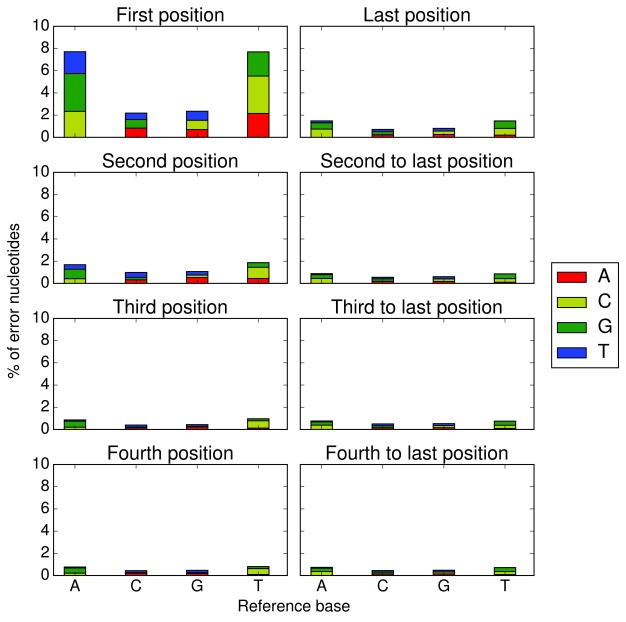
Percentage of error nucleotides at first four positions (left column) and last four positions (right column) in the first strands. RNA-seq data from GM12878 ^11^, mapped with TopHat2 v2.0.12.

Bases located either at the beginning or end of a read are particularly vulnerable to spurious base changes. The base changes at the beginning of the reads arise during first-strand cDNA synthesis using random hexamers ^14^, whereas the base changes at the end result from the read quality getting worse during sequencing and/or adapter read-through. To deal with this, base-changes in the first *N* and last *M* bases of the original read are ignored by Opossum by setting the corresponding base qualities to zero ( *’MinFlankStart’* and *’MinFlankEnd’* parameters, default=0 for both). The values for *N* and *M* can be determined by evaluating the base mismatch rates at each position of the reads in the sample as shown in Figure 1. *N* and *M* would correspond to a threshold below which the mismatch rate falls which is considered acceptable by the user. In the example, the threshold for the error rate was set to 1 percent and therefore the corresponding *’MinFlankStart’* value to 3 as the error rate has fallen below 1% at the third base position. The same applies to the last bases, with the error rate falling definitely below 1% at the third to last position, so *’MinFlankEnd’* was set to 3 as well. Opossum does not currently differentiate between first and second strands and therefore the parameter values obtained for the first strand are applied to all reads. Although the second strand should have less base mismatches ^14^, it is worth checking that the chosen parameters are in line with it as well. We have provided the code for computing base mismatch rates on GitHub.

The behavior of the *’MinFlank’* parameters depend on whether the user has set the *’SoftClipsExist’* parameter to True. If yes, then *’MinFlankStart’* and *’MinFlankEnd’* are only applied to reads containing soft clips. This is because having soft clips indicates that the mapper has had more trouble in aligning the read, and the read can exhibit a much higher base mismatch rate than a read without soft clips. Whether or not the BAM file contains reads with soft clips depends on the mapper used – for instance, by default settings, Star ^8^ is a more aggressive mapper than TopHat ^7^, tolerating many more base mismatches and marking those occurring at read ends as soft clips.

## Results

RNA-seq data from the pilot genome GM12878 ( https://www.encodeproject.org/experiments//ENCSR000COQ/, GEO accession code: GSM758559) ^11^ was used to validate the performance of Opossum. The data consisted of 26,978,818 paired-end 76 bp reads. The data was mapped with two different aligners, TopHat2 (v2.0.12) ^7^ and Star 2-pass (v2.4.2) ^8^, which have been shown to be among the best aligners for RNA-seq data ^15^. The aligned reads were then processed with Opossum, followed by variant calling with either Platypus (v0.8.1) ^5^ or GATK HaplotypeCaller (v3.4) ^9^. When using Platypus, also variants flagged as ’badReads’, ’SC’, or ’Q20’ were taken into account. The results were compared with the benchmark variant calls (v2.19) provided by GIAB (Genome in a Bottle Consortium) for NA12878 ( ftp://ftp-trace.ncbi.nlm.nih.gov/giab/ftp/release/NA12878_HG001/NISTv2.19/, ^10^). The bed file corresponding to GIAB v2.19 was used to restrict variant calls to reliable regions only.

Both precision and sensitivity were computed to evaluate the performance of each variant calling pipeline: Opossum + Platypus, Opossum + GATK HaplotypeCaller, and GATK pipeline (following its Best Practices for RNA-seq guideline, https://software.broadinstitute.org/gatk//guide/article?id=3891). Precision is defined as the fraction of true positives out of all variant calls in RNA-seq data that are supported by at least two reads (two reads is the minimum required by Platypus and GATK HaplotypeCaller by default). For evaluation purposes, those called variants that have been previously reported as RNA-editing sites ^16^ have been excluded. Sensitivity is defined as the fraction of true positives out of all variant calls in reference data (true positives + false negatives) that are supported by at least two reads in the original (deduped but otherwise unprocessed) BAM file.

Table 1 shows that pre-processing RNA-seq data with Opossum maintains high precision and improves sensitivity regardless of whether variant calling is done with GATK or Platypus. For RNA-seq data mapped with TopHat2, precision improves slightly if data is pre-processed with Opossum, while sensitivity increases by 2–3%. For data mapped with Star 2-pass, the Opossum + Platypus pipeline stands out by improving the sensitivity by more than 4%. It is also worth noting that pre-processing with Opossum slightly improves both precision and sensitivity when used in conjunction with GATK HaplotypeCaller, even though Star is recommended by GATK Best Practices and should therefore provide optimal input for the GATK variant caller.

**Table 1.** Precision, sensitivity, and runtimes for the three different variant calling pipelines.

Mapper	Variant calling pipeline	Runtime	Precision (%)	Sensitivity (%)
TopHat2	GATK Best Practices	11 h 50 min	97.04	90.08
	Opossum + GATK HaplotypeCaller	13 h 35 min	97.88	92.20
	Opossum + Platypus	5 h 40 min	97.33	92.96

Star 2-pass	GATK Best Practices	14 h 45 min	96.37	88.47
	Opossum + GATK HaplotypeCaller	15 h 35 min	96.92	89.65
	Opossum + Platypus	7 h 0 min	95.23	94.07

Using Platypus also offers a substantial reduction in runtimes compared to GATK – the runtimes fell by at least 50%. This is in line with the processing times reported in the original Platypus publication ^5^.

**Figure 2.**
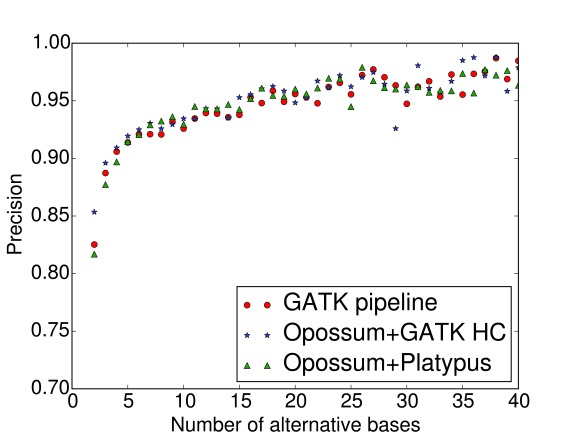
Precision as a function of the number of supporting bases. RNA-seq data mapped with TopHat2 v2.0.12. GATK HC stands for GATK HaplotypeCaller v3.4.

**Figure 3.**
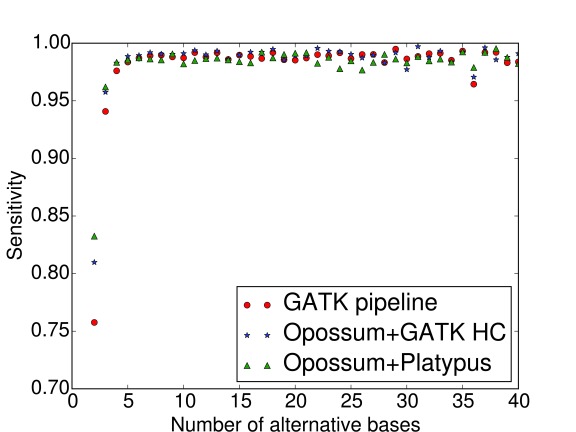
Sensitivity as a function of the number of supporting bases. RNA-seq data mapped with TopHat2 v2.0.12. GATK HC stands for GATK HaplotypeCaller v3.4.

Precision and sensitivity are presented as a function of number of supporting bases in Figure 2 and Figure 3. It can be seen that sensitivities converge rapidly to their final value: approximately four supporting reads are enough to detect a variant with a very high probability. Figure 3 also pinpoints that the superiority of the Opossum + Platypus pipeline regarding sensitivity originates from variant calls in very low-coverage areas, with only 2–3 supporting reads. According to Figure 2, precision gets to around 90% with four supporting reads and then steadily increases with higher coverage, with no major differences in the performance between the three pipelines. Both precision and sensitivity require at least two supporting reads in order to be considered in the first place.

In conclusion, the combination of Opossum + Platypus would be recommended especially in cases when the user aims for high sensitivity for SNPs, regardless of the mapper used. Moreover, Opossum + Platypus provide the best results with fastest runtimes so it is ideal when there are only limited time or computational resources available.

Having validated the capability of Opossum to process RNA-seq data for SNP detection, the next logical step would be to extend its use to detecting indels in future releases. This not only poses stricter demands on the variant caller, but also specifically on the aligner used ^17^, and has not yet been explored very much in the literature. Further compatibility will also be tested with other RNA-seq aligners (e.g. HiSat2 ^13^) and future developments of variant callers.

## Software availability

Latest source code:

https://github.com/BSGOxford/Opossum

Archived source code as at the time of publication:

https://dx.doi.org/10.5281/zenodo.223009

### License

GNU GPL v3.
