# Supplementary material for: Making the most of RNA-seq: Pre-processing sequencing data with Opossum for reliable SNP variant detection
Source: Wellcome Open Res. 2017 Mar 17;2:6. Originally published 2017 Jan 17. [Version 2] doi: 10.12688/wellcomeopenres.10501.2 (PMC5322827; doi:10.12688/wellcomeopenres.10501.2)
Supplement: Peer review history

# Peer review: Referee response for version 2

**DOI:** 10.21956/wellcomeopenres.12053.r21161

**Published:** 2017-03-21

**Authors:** Georg W. Otto

**Affiliations:** 1 University College London, London, UK

My concerns have been addressed appropriately in version 2 and I approve indexing of the article without further reservations.

I have read this submission. I believe that I have an appropriate level of expertise to confirm that it is of an acceptable scientific standard.

# Peer review: Referee response for version 1

**DOI:** 10.21956/wellcomeopenres.11317.r20032

**Published:** 2017-02-17

**Authors:** Baohong Zhang

**Affiliations:** 1 Early Clinical Development, Pfizer Worldwide Research and Development, Cambridge, MA, USA

The authors had applied some clever techniques to remove potential false mutations introduced by splicing or RNA editing and developed a pipeline which is even better than the "gold standard" GATK best practice for RNAseq according to the benchmarking. It is a good addition to the ever-growing RNAseq tool box.

 However, the author should clarify few wrong claims.

 First and foremost, "RNA-seq provides a cost-effective alternative to whole genome sequencing (WGS) for detecting genomic variants" is a wrong claim since RNAseq only cover partial of the genome where gene are expressed. The genomics coverage provided by RNAseq is different in different tissues or under various biological conditions. RNAseq only covers about 20-40% of exome. This sentence needs to be re-written or removed.

 Based on this page ( https://sequencing.qcfail.com/articles/mapq-values-are-really-useful-but-their-implementation-is-a-mess/ ), both TopHat and Star are using quite discrete mapping quality scores. The default cutoff of 40 doesn't make too much sense here. A cutoff of from 4 to 49 will create the same result. The author should point out this pitfall and propose a better scoring method for removing bad quality reads.

I have read this submission. I believe that I have an appropriate level of expertise to confirm that it is of an acceptable scientific standard.

# Peer review: Referee response for version 1

**DOI:** 10.21956/wellcomeopenres.11317.r19437

**Published:** 2017-02-09

**Authors:** Raffaele Adolfo Calogero

**Affiliations:** 1 Department of Molecular Biotechnology and Health Sciences, University of Torino, Torino, Italy

OPOSSUM prepares RNAseq data for variant calling and addresses a very important issue in the use of RNAseq for variant calling: preprocessing.

 Opossum seems to be faster than GATK and provides some improvement in sensitivity.

 In GATK RNAseq best practice, after RNAseq data preprocessing, there is Indels realignment and  base recalibration ( http://gatkforums.broadinstitute.org/gatk/discussion/3892/the-gatk-best-practices-for-variant-calling-on-rnaseq-in-full-detail). Is this part not required for variant calling after OPOSSUM preprocessing?

 Minor comment:

 This link is broken: https://github.com/luntergroup/octopu

I have read this submission. I believe that I have an appropriate level of expertise to confirm that it is of an acceptable scientific standard.

# Peer review: Referee response for version 1

**DOI:** 10.21956/wellcomeopenres.11317.r19439

**Published:** 2017-01-31

**Authors:** Georg W. Otto

**Affiliations:** 1 University College London, London, UK

Data from RNA-Seq, usually used for expression analysis, can be coopted to find DNA variants in expressed regions and sites of RNA-editing. A caveat lies in the fact that the two sources of variation can not necessarily be distinguished in a straight-forward manner, and that analyses of allele specific expression might be hampered by biases in mapping and variant calling. mRNA-levels vary by many orders of magnitude, so in order to detect variants in lowly expressed genes, the detection method has to be precise and sensitive in regions covered by only a few reads.

 Taking this into account and with the focus being restricted to expressed regions of the genome, RNA-Seq is a cost-effective alternative to whole genome sequencing. A tool that helps improving the process, by increasing precision, sensitivity and processing speed would be useful and, indeed, would make the most out of RNA-Seq. The authors show that Opossum meets these demands.

 Rather than being a variant caller itself, Opossum is basically a preprocessing pipeline to make RNA-seq reads better suited for variant calling than the original raw data. The process executed by Opossum includes:

1. Quality control and removal of spuriously mapped read-pairs.
2. Duplicate removal and solving of variant calling conflicts between read duplicates.
3. Merging of overlapping reads.
4. Splitting of intron-spanning reads.
5. Flagging of first N and last M bases to be ignored.

This is described in the manuscript in a clear and comprehensive manner.

 The authors show that there is a marked increase in sensitivity using the combination of Opossum and Platypus, compared to the GATK Best Practices Pipeline. Likewise, computation time is significantly reduced. This supports the claim that Opossum is a useful tool for variant calling of RNA-Seq data.

 There are a couple of points that remain to be addressed, though:

1. Opossum is a python script, so installation is not a problem. However, it uses samtools sort, and there is an incompatibility with samtools versions. The samtools version used to test the software (1.2) requires a file prefix for temporary files to be stated, which the Opossum code fails to do, causing an error. This should be fixed or at least the dependencies should be stated clearly.
2. It remains unexplained how much of the described improvement of sensitivity is due to Opossum processing or to Platypus variant calling (compared to GATK). We are only presented results with Opossum and Platypus in combination. Is it possible to use Platypus on RNA-seq data at all without the Opossum step? This is not discussed in the manuscript. The authors should make that point clearer.
3. As a minor remark, the sentence in the last paragraph "Having validated the capability of Opossum to detect SNPs" is not entirely accurate, since Opossum itself does not do the variant calling.

In conclusion, Opossum is a tool that is useful for a specific task in the variant calling process of RNA-Seq data. The Opossum/Platypus combination results in an increased sensitivity and reduced computation time compared to the GATK Best Practices pipeline. This is of potential benefit for researchers interested in genomic variation in expressed regions, especially in allele-specific expression, and in RNA editing. Therefore, this manuscript deserves to be indexed once the above mentioned points have been addressed.

I have read this submission. I believe that I have an appropriate level of expertise to confirm that it is of an acceptable scientific standard, however I have significant reservations, as outlined above.
